# Reviewers in 2023

**DOI:** 10.1098/rsob.240048

**Published:** 2024-03-27

**Authors:** Jon Pines

**Affiliations:** *Editor-in-Chief*

The Editors of *Open Biology* would like to thank all reviewers who contributed their time by refereeing manuscripts submitted throughout 2023. From previous feedback, we know that most reviewers value recognition of their efforts by the journal and through services, such as Web of Science Reviewer Recognition Science, which is integrated with *Open Biology*.

Under our scheme to reward peer review (https://royalsocietypublishing.org/rsob/reviewer-rewards), reviewers of *Open Biology* will be awarded tokens that can be redeemed against article processing charges (APCs) when they publish with us.

To further provide opportunities for recognition, we list the names of all reviewers who opted in. This article is made permanent and citeable by its digital object identifier (DOI). We encourage you to quote your contribution in your applications for tenure and grants or other forms of research assessment. Where you have provided it, we have included your ORCID, so your contribution can be unambiguously assigned to you.

The team really does appreciate all the work our reviewers do on behalf of the journal, and we look forward to working with you again in future.

Allen MA
Allinson S
Andreev D
Antelmann H
Arbeitman M
Avidor-Reiss T https://orcid.org/0000-0003-0918-526X
Bähler J
Balasubramaniam S
Balboula A
Barraud P https://orcid.org/0000-0003-4460-8360
Becker N
Berkmen M
Blair S
Boscaro V https://orcid.org/0000-0003-4374-1231
Botte C Y.
Bramkamp M
Bratkovič IH
Brewer G
Bruns T
Bubeck D
Buchman V
Butnariu M
Casasnovas J
Challagundla DK
Chan CX https://orcid.org/0000-0002-3729-8176
Chen S https://orcid.org/0000-0003-4565-4772
Chen T
Cho S-H
Cowan M
Crava MC https://orcid.org/0000-0003-3774-4567
Cross R
Currie S
Davis T https://orcid.org/0000-0003-4797-3152
De Felice S https://orcid.org/0000-0003-3065-3363
De Meyts P https://orcid.org/0000-0001-6214-0824
Dey G https://orcid.org/0000-0003-1416-6223
Di Cristo G
Dong B https://orcid.org/0000-0003-1616-5363
Elsässer S
Engstler M https://orcid.org/0000-0003-1436-5759
Fiedler LR
Fok E
Fukada S
Fulka H
Furlong E
Galkin A
Gamsjaeger R https://orcid.org/0000-0003-1095-2569
Ganau M https://orcid.org/0000-0002-8676-1147
Gasmi L
Gaudet R
Glueck I
Grosshans J
Guettler S
Gui F-R
Gunzl A
Gupta N
Hall M
Hamdy NM
Han C
Harada M
Hardwick K
Hauck C
Häussler U
Hernández-Morales M
Herrera SC
Hiesinger R
Higgins J https://orcid.org/0000-0001-6622-8268
Hime GR
Huber D https://orcid.org/0000-0002-7367-3244
Islas L
Jantas D
Jiang H-B
Joanna C https://orcid.org/0000-0001-7613-8127
Jontes J
Kalinina NO
Kastaniotis A
Kay RR https://orcid.org/0000-0001-9836-7967
Ke H
Kee K
Khan A
Kontrogianni-Konstantopoulos A
Kumari R https://orcid.org/0000-0001-8902-1729
Legname G
Lewis P https://orcid.org/0000-0003-4537-0489
Long M
Longdon B https://orcid.org/0000-0001-6936-1697
Lu K
Luxton GWG
Lynch J
Ma D
Ma J
Mair W
Marangoni F
Medigeshi GR
Meher PK
Merdes A http://orcid.org/0000-0002-3739-2728
Monje-Casas F
Morona R
Mura C
Murray S https://orcid.org/0000-0001-7096-1307
Nano M
Nawy S
Noreen S
Ohkura H
Paix A
Park J-B
Pascual E https://orcid.org/0000-0002-9577-840X
Patel N
Peirson S https://orcid.org/0000-0003-3653-834X
Pinali C https://orcid.org/0000-0001-9815-6940
Pinotsis N https://orcid.org/0000-0002-5096-257X
Pintard L
Plusa B
Poulin LF
Prokop A
Quashie PK
Raisman-Vozari R
Reddy A
Redondo Muñoz J
Ribeiro Fernandes H
Roithova A
Rothbächer U https://orcid.org/0000-0002-9989-6139
Roy S
Ruberti G
Saggio I
Schluckebier G https://orcid.org/0000-0003-0725-4081
Scott K
Seifert M
Sharbrough J
Sherlock G https://orcid.org/0000-0002-1692-4983
Shiga S
Silva L https://orcid.org/0000-0002-8203-4006
Simonelig M https://orcid.org/0000-0002-3731-6979
Sinigiaglia C
Spenkelink L
Spletter ML
Sprakel J
Stearns T
Stewart G
Strauss MT https://orcid.org/0000-0003-3320-6833
Strutt D
Su J https://orcid.org/0000-0002-9083-0715
Sugasawa K https://orcid.org/0000-0001-7937-4053
Swayne LA
Takaori A https://orcid.org/0000-0001-7678-4284
Tapias V
Tegeder I
Terai T
Tocher D
Tomášek O https://orcid.org/0000-0002-2657-5916
Tooze S
Vagnarelli P https://orcid.org/0000-0002-0000-2271
Van Eeden F
Vecsey CG https://orcid.org/0000-0003-1980-5354
Velthuis AJWT
Vincent C https://orcid.org/0000-0002-4812-4913
Vogt K https://orcid.org/0000-0002-8763-342X
Wang J https://orcid.org/0000-0002-1317-6457
Wang X
Weber T
Weedall G
Wegrzyn G
Westermann S
Wirtshafter H
Wisden W
Xian H
Xie Z
Xu X
Yakoby N
Yang C
Yu H
Zhao X
Zhou X https://orcid.org/0000-0002-4551-0608
Zinn K
Zuloaga K

